# PEGylated Carbon Nanotubes Impair Retrieval of Contextual Fear Memory and Alter Oxidative Stress Parameters in the Rat Hippocampus

**DOI:** 10.1155/2015/104135

**Published:** 2015-02-09

**Authors:** Lidiane Dal Bosco, Gisele E. B. Weber, Gustavo M. Parfitt, Karina Paese, Carla O. F. Gonçalves, Tiago M. Serodre, Clascídia A. Furtado, Adelina P. Santos, José M. Monserrat, Daniela M. Barros

**Affiliations:** ^1^Programa de Pós-Graduação em Ciências Fisiológicas-Fisiologia Animal Comparada, Instituto de Ciências Biológicas, Universidade Federal do Rio Grande, 96203-900 Rio Grande, RS, Brazil; ^2^Programa de Pós-Graduação em Ciências Farmacêuticas, Faculdade de Farmácia, Universidade Federal do Rio Grande do Sul, 90610-000 Porto Alegre, RS, Brazil; ^3^Laboratório de Química de Nanoestruturas de Carbono, Centro de Desenvolvimento da Tecnologia Nuclear, C.P. 941, 31270-901 Belo Horizonte, MG, Brazil

## Abstract

Carbon nanotubes (CNT) are promising materials for biomedical applications, especially in the field of neuroscience; therefore, it is essential to evaluate the neurotoxicity of these nanomaterials. The present work assessed the effects of single-walled CNT functionalized with polyethylene glycol (SWCNT-PEG) on the consolidation and retrieval of contextual fear memory in rats and on oxidative stress parameters in the hippocampus. SWCNT-PEG were dispersed in water at concentrations of 0.5, 1.0, and 2.1 mg/mL and infused into the rat hippocampus. The infusion was completed immediately after training and 30 min before testing of a contextual fear conditioning task, resulting in exposure times of 24 h and 30 min, respectively. The results showed that a short exposure to SWCNT-PEG impaired fear memory retrieval and caused lipid peroxidation in the hippocampus. This response was transient and overcome by the mobilization of antioxidant defenses at 24 h. These effects occurred at low and intermediate but not high concentration of SWCNT-PEG, suggesting that the observed biological response may be related to the concentration-dependent increase in particle size in SWCNT-PEG dispersions.

## 1. Introduction

The unique physical properties and morphological characteristics of carbon nanotubes (CNT) have made them interesting for many biomedical applications, including therapeutic strategies for the treatment of central nervous system injuries [1–3]. However, few studies reported the effects of CNT on the central nervous system of animals. Studies of systemically exposed mice have shown cognitive deficits and increased oxidative stress levels in brain [4], although these effects were not observed in fishes [5, 6]. It should be considered that systemic administration of CNT likely results in different biodistribution and toxicity profiles based on the type of nanomaterial, the route of administration, and the animal model used [7–9]. Thus, an alternative method for studying the interactions between CNT and neural tissue is the direct administration of the nanomaterial into the brain by stereotactic injection [10, 11].

Exposure to CNT is related to increased production of reactive oxygen species (ROS) and depletion of the cellular antioxidant defenses, culminating in damage to the cellular components, as lipids, proteins, or DNA [4, 12, 13]. The ability of CNT to induce oxidative stress in brain tissue is a cause of concern, since this condition has been implicated in the physiopathology of neurodegenerative disorders [14–16]. Functionalization of CNT with polyethylene glycol (PEG) has been studied as a strategy to prevent neurotoxicity [17] and optimize neurite-outgrowth-promoting properties of CNT [3]. However, more studies regarding the effects of PEGylated CNT on brain and behavior of animals are required prior to their applications in neurology.

The present study aimed to investigate the effects of single-walled CNT functionalized with polyethylene glycol (SWCNT-PEG) directly infused into the hippocampus, a brain structure critical for episodic and spatial memory that plays an important role in processing contextual information [18]. Contextual fear conditioning task was employed to evaluate the consolidation and retrieval of contextual fear memory. Oxidative stress parameters were determined by measuring reactive oxygen species (ROS) levels, lipid peroxidation (LPO), total antioxidant capacity against peroxyl radicals (ACAP), glutathione (GSH) content, and glutamate-cysteine ligase (GCL) activity in the rat hippocampus. The contributions of physicochemical characteristics to the effects of SWCNT-PEG dispersions in the hippocampus were discussed.

## 2. Material and Methods

### 2.1. Chemicals

Tris-HCl (Trizma hydrochloride), ethylenediaminetetraacetic acid (EDTA), 2,2′-azobis(2-methylpropionamidine) dihydrochloride (ABAP), glutathione (GSH), adenosine triphosphate (ATP), glutamate, borate, serine, cysteine, cumene hydroperoxide (CHP), and xylenol orange were purchased from Sigma-Aldrich (St. Louis, MO, USA). KCl, MgCl_2_, and FeSO_4_ were purchased from Labsynth (Diadema, SP, Brazil). 2′,7′-Dichlorofluorescein-diacetate (H_2_DCF-DA) and naphthalene-2,3-dicarboxaldehyde (NDA) were purchased from Molecular Probes (Eugene, OR, USA). Biuret protein assay kit was purchased from Doles (Goiânia, GO, Brazil).

### 2.2. Preparation of SWCNT-PEG Dispersions

Single-walled carbon nanotubes (SWCNT) synthesized by electric arc method and functionalized with polyethylene glycol (PEG, Mw = 600 Da) were purchased from Sigma-Aldrich (Lot MKBC 9435). The dispersion protocol of SWCNT-PEG was adapted from the procedure of Kalinina et al. [19] and employed several steps of mechanical disintegration and centrifugation. SWCNT-PEG dispersed in water at 3 mg/mL were subjected to ultrasonication for 48 h using a bath sonicator (Cole Parmer, model 08895-50, ~40 kHz) and subsequently placed in a high-shear rotor-stator mixer (IKA Labortechnik, Ultra-Turrax T8) for 90 min. The mixture was centrifuged (Eppendorf, 5417C) for 30 min at 5,000 g to remove large particles and agglomerates and the supernatant was carefully removed, diluted four times, and placed in an ultracentrifuge (Sorvall, Ultra Pro 80) for 1 h at 170,000 g to remove any excess unbound PEG, nontubular carbon and other impurities. Next, the resulting pellet was resuspended in water to give a dispersion concentration of approximately 2 mg/mL and then subjected again to ultrasonication for 4 h, shear mixing for 30 min, and centrifugation for 30 min at 5,000 g. The final concentration of SWCNT-PEG in the final dispersion was determined as previously described by Weber et al. [9] and was estimated to be 2.1 ± 0.2 mg/mL based on the intensity of light absorption at 700 nm. Dilutions of the final dispersion were made by adding ultrapure water to obtain concentrations of 0.5 and 1.0 mg/mL. After addition of the water, dilutions underwent an ultrasonication for 30 min.

### 2.3. Physicochemical Characterization

The purity, size distribution, and surface coating of the SWCNT-PEG sample were previously described by our group [9]. In this work, SWCNT-PEG dispersions were characterized by low resolution (LR) transmission electron microscopy (TEM) (FEI Tecnai G2-Spirit 120 kV microscope) before completion and at the end of the dispersion protocol. The final dispersion of SWCNT-PEG was also characterized by high-resolution (HR) TEM (FEI Tecnai G2-20 Super-Twin 200 kV microscope) and Raman spectroscopy. Raman spectra were obtained using a modular Raman system (Horiba Jobin Yvon, RMS-550) with an excitation wavelength of 514 nm. Zeta potential of SWCNT-PEG dispersions at 0.5, 1.0, and 2.1 mg/mL was determined by electrophoretic light scattering (Zetasizer Nano ZS system, Malvern Instruments) after diluting the original dispersion 10 times following a 30 min bath sonication, filtering through a 0.45 *μ*m membrane, and adjusting the ionic strength to 10 mM using NaCl. Size distribution, polydispersity index (PDI), and hydrodynamic particle diameter (z-average) were analyzed by dynamic light scattering (DLS) (Zetasizer Nano ZS system, Malvern Instruments). Z-average and PDI were calculated using a cumulant analysis. The hydrodynamic diameter of SWCNT-PEG measured by DLS corresponds to the equivalent hydrodynamic diameter of a sphere that has the same translational diffusion coefficient. Thus, the data obtained do not correspond to a single dimension (length or diameter) of the nanomaterial, but rather to a combined value of both dimensions.

### 2.4. Animals

Wistar male rats (2-3 months of age, weight 250–320 g) were obtained from a breeding colony at the Universidade Federal do Rio Grande (RS, Brazil) and randomly housed in polypropylene cages of 41 × 34 × 16 cm in number of five rats per cage with free access to water and food. The animals were kept under a 12 h light/dark cycle and constant temperature (23 ± 1°C) and were frequently handled to avoid neophobia. All animal procedures were approved by the Ethics Committee for Animal Use of the Universidade Federal do Rio Grande (FURG; Permit Number: P029/2011).

### 2.5. Experimental Design

After a week of acclimation, the rats were bilaterally implanted with stainless steel guide cannulae (22-gauge, 9 mm in length) in the dorsal CA1 region of hippocampus. Stereotaxic coordinates were 4.3 anterior, 3.0 lateral, and 1.8 ventral according to the atlas by Paxinos and Watson [20]. After 48–72 h of recovery from surgery, rats were distributed randomly into treatment groups (*n* = 10–12). SWCNT-PEG dispersions of 0.5, 1.0, or 2.1 mg/mL or a 0.9% NaCl solution (control group) was infused using 27-gauge injection needles inserted into each guide cannula and connected by polyethylene tubing to a Hamilton microsyringe. All infusions were 1 *μ*L in volume and were completed one hemisphere at a time. The infusions were performed immediately after the training session or 30 min before testing of a contextual fear conditioning task, resulting in the exposure times of 24 h and 30 min, respectively.

### 2.6. Contextual Fear Conditioning Task

The contextual fear conditioning (CFC) apparatus consisted of a box (dimensions 28 × 26 × 23 cm) with three aluminum walls, one Plexiglas front wall, and a floor made of parallel stainless steel bars connected to a shock scrambler deliver apparatus (shock generator, Insight Scientific Equipment, Brazil). The CFC task was performed as previously described by Bekinschtein et al. [21]. Briefly, the training session consisted of three consecutive single electric foot-shocks (1 sec duration, 0.7 mA intensity) at 10 sec intervals. The test session was performed twenty-four hours after training. During the 5 min test, animals were monitored for freezing (absence of any movement except breathing). Both training and test sessions were performed between 8:00 and 12:00 a.m., and after each session the floor and walls of the CFC apparatus were cleaned with 70% ethanol. The results are expressed as the percentage of time spent in freezing during a 5 min period.

### 2.7. Tissue Dissection and Sample Preparation

At the end of the CFC test, all animals were euthanized by decapitation. The hippocampi were quickly dissected and stored at −80°C or immediately homogenized (1 : 5 w/v) in 40 mM ice-cold Tris-HCl buffer (pH 7.4) for ROS measurements. For antioxidant capacity against peroxyl radicals (ACAP), glutamate cysteine ligase (GCL) activity, and glutathione (GSH) content analysis, hippocampi were thawed on ice and homogenized in a buffer containing 100 mM Tris-HCl, 2 mM EDTA, and 5 mM MgCl_2_ (pH 7.75). Tissue homogenates were centrifuged at 20,000 g at 4°C for 20 min. The total protein content was measured in the supernatant with the Biuret method using a microplate absorbance reader (BioTek LX 800). The final protein concentration was adjusted to 3 mg/mL. For LPO analysis, hippocampi were homogenized (1 : 15 w/v) in 100% ice-cold methanol and centrifuged at 1000 g for 10 min at 4°C. Supernatants were used in all assays.

### 2.8. Measurement of Reactive Oxygen Species Levels

Reactive oxygen species (ROS) levels in rat hippocampus were determined by measuring the oxidation of H_2_DCF-DA as previously described for brain tissue analysis [22, 23]. Briefly, the samples were placed in a reaction buffer (pH 7.2) containing 200 mM KCl, 30 mM HEPES, 1 mM MgCl_2_, and 16 *μ*M H_2_DCFDA. Formation of the oxidized fluorescent product dichlorofluorescein (DCF) was monitored with a fluorescence microplate reader (485 nm excitation/520 nm emission; Victor 2, Perkin Elmer). Data were collected every 5 min for a total of 30 min. ROS levels were calculated by integrating the fluorescent units (FU) over time and fitting the data to a second order polynomial function. Two parallel experiments were conducted and values of FU were normalized to a percentage of the control group.

### 2.9. Total Antioxidant Capacity against Peroxyl Radicals

Determination of total antioxidant capacity against peroxyl radicals (ACAP) was performed as described by Amado et al. [24]. The assay employed ROS quantification using H_2_DCF-DA (40 *μ*M final concentration) in hippocampal samples treated with or without a peroxyl radical generator (ABAP, 4 mM). DCF formation was recorded by a fluorescence microplate reader (485 nm excitation/520 nm emission; Victor 2, Perkin Elmer). Data were collected every 5 min for a total of 30 min. The relative difference between ROS area with and without ABAP was calculated and the inverse of the relative difference was considered as a measure of antioxidant capacity.

### 2.10. Glutathione Reduced Content and Glutamate-Cysteine Ligase Activity

Glutathione reduced (GSH) content and glutamate-cysteine ligase (GCL) activity were determined according to the methods of White et al. [25]. This technique is based on the reaction of naphthalene-2,3-dicarboxaldehyde (NDA) with GSH or *γ*-glutamylcysteine (*γ*-GC) to form cyclic products that are highly fluorescent. Further assay details were outlined by da Rocha et al. [26]. NDA-GSH fluorescence was measured (485 nm excitation/530 nm emission) using a fluorescence microplate reader (Víctor 2, Perkin Elmer). GSH content was expressed in *μ*M per mg of protein and GCL activity in *μ*M of GCL h^−1^ per mg of protein.

### 2.11. Measurement of Lipid Peroxidation

Lipid peroxide levels were measured using a spectrophotometric ferrous oxidation/xylenol orange (FOX) modified method as previously described by Monserrat et al. [27] except for adjustments in the time of incubation and sample dilution which were based on work by de Aguiar et al. [22]. This method is based on the oxidation of Fe(II) under acidic conditions, and the quantification of lipid hydroperoxides was obtained using 0.1 mM CHP as a standard. The assay was performed in a microplate using 5 *μ*L of sample volume or 5 *μ*L of methanol as negative control. The absorbance (550 nm) was determined using a microplate reader (BioTek LX 800). Lipid peroxidation (LPO) was expressed as nM CHP per gram of wet tissue.

### 2.12. Statistical Analysis

Statistical analysis and graph creation was carried out in GraphPad Prism 5.0 (GraphPad Software, Inc. La Jolla, CA, USA). The hydrodynamic size, polydispersity index, and zeta-potential data were analyzed using a one-way statistical analysis of variance (one-way ANOVA) followed by a Tukey post-hoc test. Biochemical and behavioral data were analyzed using a one-way ANOVA followed by the Newman-Keuls multiple comparisons test. Normality and variance homogeneity were verified. A *P* < 0.05 was considered statistically significant.

## 3. Results

### 3.1. SWCNT-PEG Characterization

**Figure 1:**
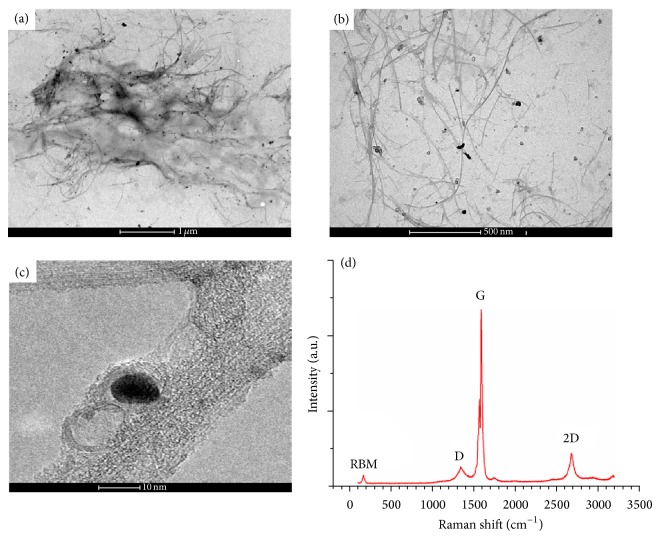
Low-resolution transmission electronic microscopy images of SWCNT-PEG dispersion after dispersed by (a) 48 h ultrasonication and (b) after the combination of ultrasonication and high-shear mixing. High-resolution transmission electronic microscopy image (c) and Raman spectra (d) of the final dispersion.

By LRTEM we observed that SWCNT were completely enveloped by the polymer and formed large aggregates that could be separated into fibers after the combination of ultrasonication and high shear mixing (Figures 1(a) and 1(b)). Besides these fibers, impurities and isolated polymer plates were also observed. HRTEM analysis revealed that SWCNT were functionalized in the form of bundles and even after the dispersion protocol was not possible to completely break down into individual tubes (Figure 1(c)). The Raman spectra of the SWCNT-PEG presented a well-defined and intense G band and radial breathing mode (RBM), even after the dispersion protocol (Figure 1(d)). This result confirmed that not all tubes were functionalized; that is, the functionalization mainly occurred on the SWCNT bundle surface rather than on individual nanotubes.

**Figure 2:**
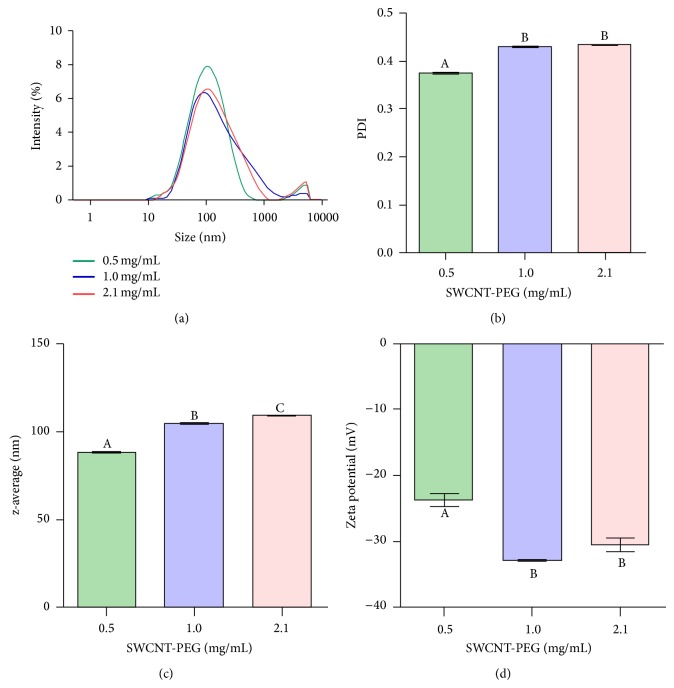
Intensity based particle size distribution (a), polydispersity index (PDI) (b), z-average (c), and zeta-potential (d) of SWCNT-PEG dispersions. Different letters indicate a significant difference (*n* = 3; *P* < 0.05).

The DLS analysis revealed a bimodal particle size distribution (Figure 2(a)) with a smaller-sized mode between 10 and 1000 nm and a larger-sized mode between 1000 and 10000 nm. The heterogeneous particle size distribution observed in this study was confirmed using PDI analysis (Figure 2(b)), which provides a measure of the broadness of the particle size distribution. PDI values of SWCNT-PEG dispersions were in the range from 0.373 to 0.436, and a lower PDI value was observed for the 0.5 mg/mL dispersion. The z-average revealed a concentration dependent increase in the particle size of SWCNT-PEG dispersed in water (Figure 2(c)). The zeta potential values were between −23 and −33 mV (Figure 2(d)) and did not correlate with the mean particle size of SWCNT-PEG dispersions.

### 3.2. Behavioral Parameter

**Figure 3:**
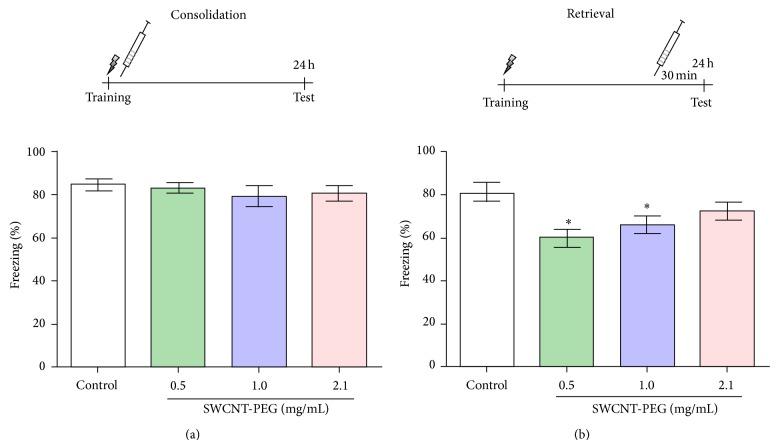
The effects of SWCNT-PEG dispersions on contextual fear memory. The infusion was completed immediately after the training session (a) and 30 minutes before the test (b). Data represent the mean values ± SEM (*n* = 10–12). ^*^Significant difference from the control group (*P* < 0.05).

As shown in Figure 3(a), SWCNT-PEG dispersions infused immediately after the training session did not affect fear memory consolidation. The animals treated with SWCNT-PEG dispersions presented a similar freezing time when compared with the control group, revealing that these animals could discriminate fear context. On the other hand, infusions of SWCNT-PEG dispersions at 0.5 and 1.0 mg/mL that occurred 30 min before the test session caused a deficit in the retrieval of fear memory (Figure 3(b)). This impairment was not observed in the group that received the SWCNT-PEG dispersion at 2.1 mg/mL.

### 3.3. ROS Levels

**Figure 4:**
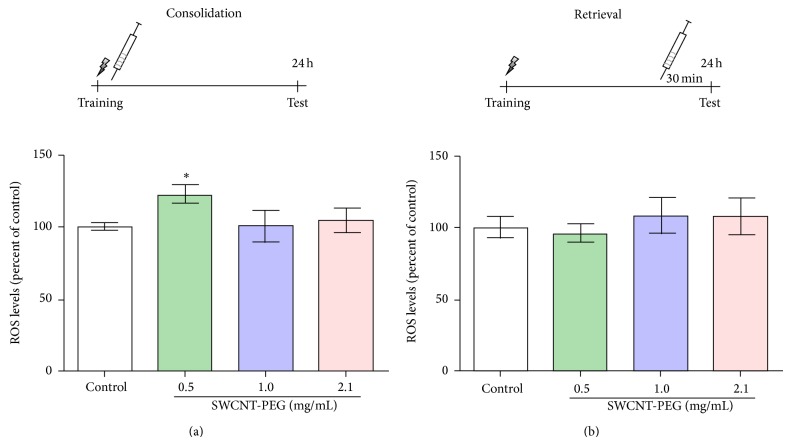
Levels of reactive oxygen species (ROS) in the rat hippocampus 24 h (a) and 30 min (b) after the infusion of SWCNT-PEG dispersions. Data represent the mean ± standard SEM (*n* = 4–6). ^*^Significant difference from the control group (*P* < 0.05).

As shown in Figure 4(a), there was a significant increase in ROS levels in the hippocampus 24 h after the infusion of SWCNT-PEG dispersions at 0.5 mg/mL. This increase was approximately 23% above the average of the control group. There was no significant difference in ROS generation between SWCNT-PEG dispersions and control group 30 min after the infusion treatment (Figure 4(b)).

### 3.4. ACAP Determination

**Figure 5:**
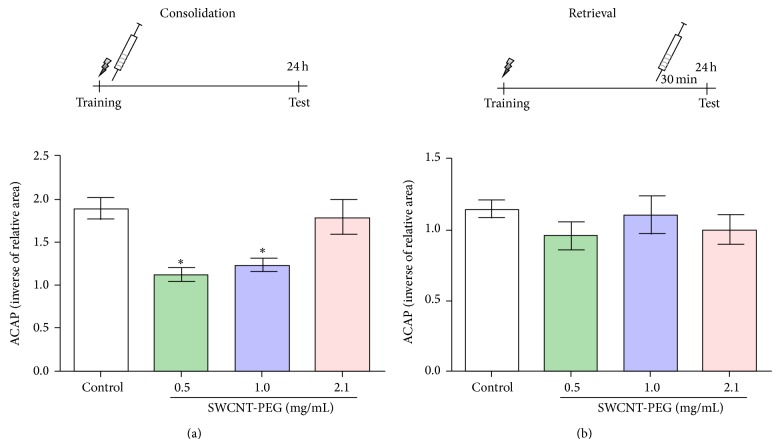
Effects of SWCNT-PEG dispersions on the antioxidant capacity against peroxyl radicals (ACAP) in the rat hippocampus 24 h (a) and 30 min (b) after treatment infusion. Data represent the mean ± standard SEM (*n* = 4–6). ^*^Significant difference from the control group (*P* < 0.05).

Hippocampal ACAP was significantly reduced 24 h after infusion of SWCNT-PEG dispersions at 0.5 and 1.0 mg/mL (Figure 5(a)). The decrement in ACAP was 35% and 30% in the groups receiving the dispersions of 0.5 and 1.0 mg/mL, respectively, when compared with the control group. There was no difference in ACAP among the experimental groups 30 min after treatment (Figure 5(b)).

### 3.5. GSH Content and GCL Activity

**Figure 6:**
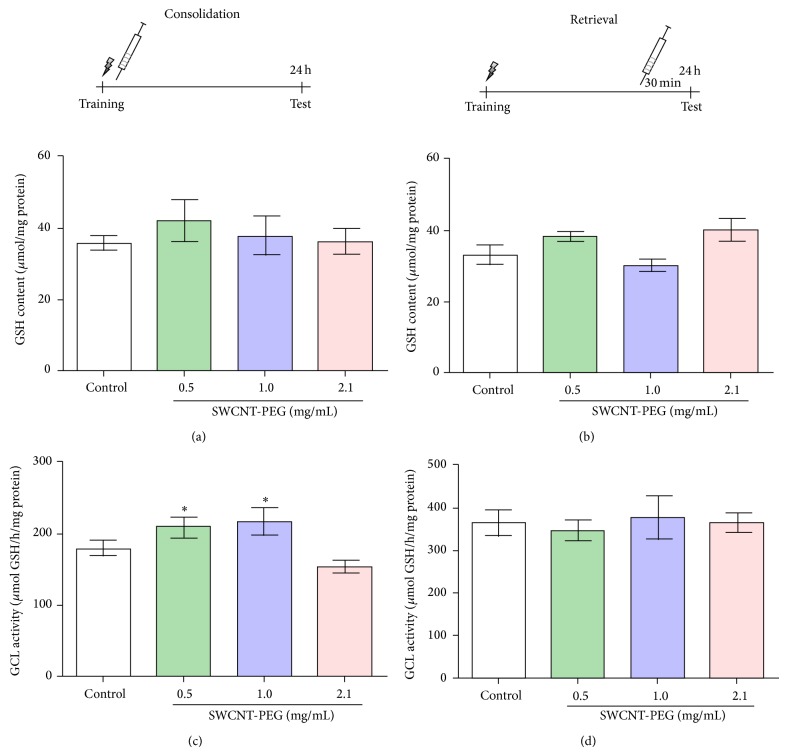
Glutathione (GSH) content 24 h (a) and 30 min (b) after intrahippocampal infusion of SWCNT-PEG dispersions. Activity of glutamate cysteine-ligase (GCL) 24 h (c) and 30 min (d) after infusion of SWCNT-PEG dispersions. Data represent mean ± standard SEM (*n* = 4–6). ^*^Significant difference from the control group (*P* < 0.05). ^*^Significant difference from SWCNT-PEG 2,1 mg/mL (*P* < 0.05).

The results of GSH content and GCL activity are presented in Figure 6. GSH content of the hippocampus was unaltered at both 30 min and 24 h after injection for the SWCNT-PEG groups (Figures 6(a) and 6(b)). A significant increase was found in GCL activity 24 h after the infusion of SWCNT-PEG dispersions at 0.5 and 1.0 mg/mL (Figure 6(c)). There was no difference in GCL activity among the treatments 30 min after infusion (Figure 6(d)).

### 3.6. LPO Measurement

**Figure 7:**
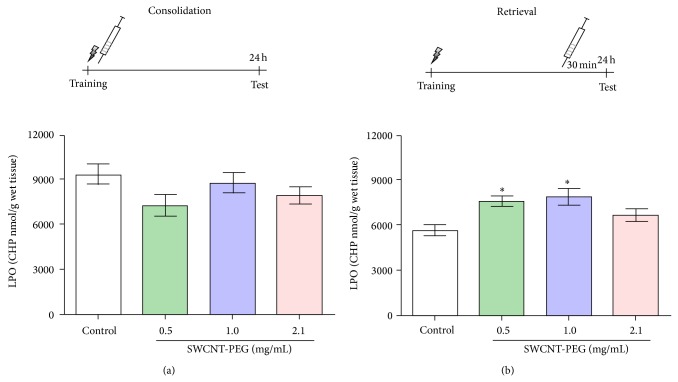
Effects of SWCNT-PEG dispersions on lipid peroxidation (LPO) 24 h (a) and 30 min (b) after treatment infusion. Data represent the mean ± standard SEM (*n* = 4–6). ^*^Significant difference from the control group (*P* < 0.05).

Lipid hydroperoxide levels in the hippocampus were unaltered 24 h after SWCNT-PEG infusion (Figure 7(a)). However, there was a significant increase in LPO 30 min after the infusion of SWCNT-PEG dispersions at 0.5 and 1.0 mg/mL (Figure 7(b)). The increase was approximately 34% and 39% for SWCNT-PEG dispersions at 0.5 mg/mL and 1.0 mg/mL, respectively, when compared with the control group.

## 4. Discussion

Neurobehavioral studies are of great importance in toxicological research because they assess the functional integrity and latent damage of the nervous system and the mechanism of action behind chemically induced neurotoxicity [28, 29]. There are a limited number of studies that have assessed neurobehavioral changes after CNT exposure, despite the growing interest in nanodevices based on CNT for applications in neuroscience. Here, we used stereotactic surgery to infuse SWCNT-PEG dispersions into the rat hippocampus and then evaluated changes in two important stages of fear memory processing.

The hippocampus is one of the most sensitive brain regions to oxidative stress [30], particularly the CA1 subregion of the dorsal hippocampus, which is highly vulnerable to oxidative stress due its high demand for reactive species as signaling molecules and the lower ATP production when compared to resistant neurons [31]. Moreover, CA1 subregion is required for the encoding, consolidation, and retrieval of contextual memories [32, 33]. The occurrence of cognitive deficits accompanied by oxidative damage and histopathological changes in hippocampus has been reported after the exposure to different nanoparticles [34, 35], including CNT [4]. In this work, SWCNT-PEG dispersions at 0.5 and 1.0 mg/mL induced lipid peroxidation in the hippocampus and impaired memory retrieval 30 min after infusion. The fact that ROS levels remained almost constant at this time point suggests that SWCNT-PEG reacted probably with unsaturated lipids, leading to lipid peroxidation. This oxidative damage induced by SWCNT-PEG probably culminated in the impairment of memory retrieval.

On the other hand, the infusion of SWCNT-PEG dispersions at 0.5 and 1.0 mg/mL did not result in oxidative damage or memory impairment 24 h after exposure but decreased the antioxidant capacity in the hippocampus at this time, although GSH content was unaltered. Besides these changes, there was an increase in ROS levels in the dispersion at low SWCNT-PEG concentration, which did not result in lipid peroxidation or impairment in memory consolidation. We suggest that this antioxidant mobilization may have contributed to reduce oxidative stress levels in the hippocampus. According to the hierarchical oxidative stress model proposed by Xiao et al. [36], the lowest levels of oxidative stress can induce cytoprotective responses that include expression of proteins involved in antioxidant defense. So, the antioxidant mobilization may have prevented the persistence of oxidative damage in the hippocampus after 24 h.

The biological response observed in this study occurred at low and intermediate but not high concentration of SWCNT-PEG dispersions, suggesting that the effects may be due to a concentration-dependent increase in hydrodynamic diameter. Thus, it is reasonable to propose that the effects of the nanomaterial in the hippocampus can be related to other physicochemical characteristics, including size, agglomeration state, and surface chemistry [37–39]. We used a DLS technique to determine the hydrodynamic properties of the CNT, including particle diameter and size distribution [40, 41].

The particle size distribution of SWCNT-PEG dispersions was a bimodal curve, indicating the presence of well-dispersed SWCNT-PEG and their agglomerates. This finding supports previously reported data on CNT dispersions [42–44]. It was observed that PDI values and z-average positively correlated with the concentration of SWCNT-PEG dispersions, suggesting a higher agglomeration tendency with larger concentrations of CNT. The concentration-dependent change in particle size was previously demonstrated for nanomaterials of different compositions [39].

It has been demonstrated that micron-sized agglomerates of SWCNT are more cytotoxic [38, 45, 46] than better dispersed SWCNT bundles. On the other hand, micron-sized agglomerates of CNT at high concentrations may have reduced toxicity because of the decrease in the reactive surface area, limiting their translocation and interaction with cells [47]. In agreement with these observations, Hirano et al. [48] reported a concave dose response curve in the cell viability of multiwalled CNT (MWCNT) exposed cells. The agglomeration of the nanomaterial occurred at high concentrations and it was suggested that this accumulation may have reduced MWCNT-cells interactions and prevented toxicity [48].

The effects of SWCNT-PEG on memory retrieval and oxidative stress observed in this study were produced by dispersions with a mean particles size of 88.1 and 104.8 nm, but not 108.9 nm. Although the difference in mean particle size between the dispersions at 1.0 and 2.1 mg/mL appears to be too small to justify distinct biological responses, it should be noted that the dispersion with 2.1 mg/mL SWCNT-PEG showed the most heterogeneous particle size distribution. The infusion of the dispersion at 2.1 mg/mL possibly resulted in few small-sized particles that were able to interact with hippocampal cells and cause neurotoxic effects. Thus, the adverse interactions of SWCNT-PEG with cellular targets may be facilitated by the smaller particles present in the dispersions at low and intermediate concentrations.

In addition to particle size, the surface functionalization of CNT may be an important factor for toxicity because it can modify water solubility, agglomeration state, and surface charge of these nanomaterials [49]. The zeta potential is an indicator of the magnitude of electrostatic interactions between colloidal particles, and its measurement has been used to determine the density of acidic sites on the surface of CNT [50, 51]. The negative zeta potential values found for SWCNT-PEG dispersions indicate the presence of multiple unreacted carboxyl acid groups (-COOH).

Functionalization by carboxylation decreased CNT toxicity* in vitro* [52] and* in vivo* [8]. However, it was also demonstrated that PEGylation of free carboxyl groups mitigated the cytotoxicity of shorter MWCNT with a high surface carboxyl density [53]. In this study, the dispersions of SWCNT-PEG at 1.0 and 2.1 mg/mL presented similar zeta potential values but had distinct effects on fear memory retrieval and oxidative stress parameters. It was unclear if a negative zeta potential affected dispersibility and subsequent toxicity of SWCNT-PEG; thus, more studies are required to ascertain the contribution of carboxyl groups and zeta potential in the biological response to CNT.

In conclusion, our results showed that SWCNT-PEG caused distinct behavioral and biochemical changes based on the time of their infusion in the hippocampus and particle size of the SWCNT-PEG agglomerates. A short-term exposure to SWCNT-PEG was able to impair memory retrieval and cause lipid peroxidation in the rat hippocampus. However, a longer exposure to the nanomaterial resulted in antioxidant mobilization, overcoming the oxidative damage. These effects occurred in the dispersions of SWCNT-PEG that presented smaller particle size. The results of this work have important implications for the safety assessment of CNT, particularly with regard to the influence of physicochemical characteristics and temporal variations on the biological responses to nanomaterial exposure. Notwithstanding, further studies on the interactions between SWCNT-PEG and neural tissue are of great importance considering the potential use of these nanomaterials in neurology.
